# Safety and efficacy of oxybutynin in patients with hyperhidrosis: systematic review and meta-analysis of randomized controlled trials

**DOI:** 10.1007/s00403-023-02587-5

**Published:** 2023-03-04

**Authors:** Mohamed El-Samahy, Adel Mouffokes, Marwa M. Badawy, Sarah Amro, Taha Fayad, Omar Ahmed Abdelwahab

**Affiliations:** 1 Medical Research Group of Egypt, Cairo, Egypt; 2 Faculty of Medicine, Zagazig University, Zagazig, Egypt; 3 Faculty of Medicine, University of Oran, Ahmed Ben Bella 1, Oran, Algeria; 4 Faculty of Medicine, October 6 University, Giza, Egypt; 5 Al-Najah National University, Nablus, Palestine; 6 Faculty of Oral and Dental Medicine, Sinai University, Al-Arish, North Sinai, Egypt; 7 Faculty of Medicine, Al-Azhar University, Cairo, Egypt

**Keywords:** Hyperhidrosis, Oxybutynin, Excess sweating

## Abstract

**Background:**

Sweating is a physiologic mechanism of human thermoregulation. Hyperhidrosis is defined as a somatic disorder where the sweating is exaggerated in an exact area because the sweat glands are hyperfunctioning. It negatively affects the quality of life of the patients. We aim to investigate patient satisfaction and the effectiveness of oxybutynin in treating hyperhidrosis.

**Methods:**

We prospectively registered the protocol of this systematic review and meta-analysis on PROSPERO (CRD 42022342667). This systematic review and meta-analysis were reported according to the PRISMA statement guidelines. We searched three electronic databases (PubMed, Scopus, Web of Science) from inception until June 2, 2022, using MeSH terms. We include studies comparing patients with hyperhidrosis who received oxybutynin or a placebo. We assessed the risk of bias using the Cochrane risk of bias assessment tool (ROB2) for randomized controlled trials. The risk ratio was calculated for categorical variables, and the mean difference was calculated for continuous variables using the random effect model with 95% confidence intervals (CI).

**Results:**

Six studies were included in the meta-analysis, with a total of 293 patients. In all studies, patients were assigned to receive either Oxybutynin or Placebo. Oxybutynin represented an HDSS improvement (RR = 1.68 95% CI [1.21, 2.33], *p* = 0.002). It also can improve the quality of life. There is no difference between oxybutynin and placebo regarding dry mouth (RR = 1.68 95% CI [1.21, 2.33], *p* = 0.002).

**Conclusion:**

Our study suggests that using oxybutynin as a treatment for hyperhidrosis is significant and needs to be highlighted for clinicians. However, more clinical trials are needed to grasp the optimum benefit.

**Supplementary Information:**

The online version contains supplementary material available at 10.1007/s00403-023-02587-5.

## Introduction

Sweating is a physiologic mechanism of human thermoregulation [1]. Hyperhidrosis is defined as a somatic disorder where the sweating is exaggerated in an exact area because the sweat glands are hyperfunctioning [1, 2]. The eccrine glands are concentrated in the regions such as the face, palms, axilla, and soles; therefore, these areas are mostly associated with hyperhidrosis [3]. The acetylcholine negative feedback loop is likely impaired in these patients, which can clarify how a physiologic response can change into pathological [4]. The prevalence is approximately 3% of this disorder in the United States [1]. Hyperhidrosis is classified into two types: primary and secondary, and the treatment and management can be different for each type. The primary disease (with no known cause) has more localized symptoms and is present in life earlier. The secondary disease typically presents because of side effects of systemic disorders or medications, especially neurologic [4, 5]. The diagnosis is based mainly on clinical bases, tests, and grading scales that are available to help in determining the localization and the severity [6]. The starch-iodine and gravimetry tests are usually used to determine the amount of sweat produced by specific area and localize the sites which need treatment [7]. For example, more than 50 mg of sweat per minute in the axillary area is considered hyperhidrosis [8]. The commonest grading scale used to assess the impact of sweating on the patient life and the response to treatment is the dermatology life quality index (DLQI) [9]. If a secondary cause is suspected, we may need laboratory work-up to rule out diabetes mellitus, neurologic disorder, infection, hyperthyroidism, or a medication adverse effect [3]. The treatment of secondary hyperhidrosis is treating the underlying cause or stoppage of the drug causing it [3].

Hyperhidrosis can lead to social, emotional, occupational, and psychological impairment [1]. Given the significant effect on the quality of life, identifying effective treatments is a research priority [10–12] No evidence has proved that either women or men are at increased risk [1]. The most commonly affected area is the palmar region [3]. Many therapy modalities are available: oral, topical, and injectable drugs, tap water iontophoresis, or more invasive medical treatments (e.g., suction curettage, laser therapy, endoscopic transthoracic sympathectomy, and microwave thermolysis) [13]. Over-the-counter aluminum chloride hexahydrate 20% is considered the first line of treatment for the disorder; it is given for 3 to 4 nights, then nightly as needed [14]. Patients often become intolerant of it in the long run. Furthermore, skin irritation can happen [15].

All topical agents can result in skin sensitization, and some, like potassium permanganate and tannic acid, can also lead to skin discoloration [14]. These agents can limit sweating by denaturing keratin and hence, occluding the sweat glands' pores. The duration of the effect is concise. Aluminum chloride gel can manage axillary sweating. While it does work, it is a potent irritant [14].

Botulinum toxin injection is the most studied hyperhidrosis therapy and demonstrates consistent improvement in sweat production as measured in the axillae and palms [8, 16]. It can be considered first- or second-line therapy for hyperhidrosis affecting the face, palms, axilla, soles, or face [8, 17]. Botulinum toxins bind synaptic proteins, which block the acetylcholine release from the cholinergic neurons, which innervate the eccrine sweat glands [18]. However, it is not often used because of localized numbness, weakness, and fears around its effectiveness; moreover, it causes pain [19].

Oxybutynin is an anticholinergic drug used to treat urinary frequency, incontinence, and overactive bladder [20]. Hyperhidrosis was originally linked to the antimuscarinic effects of oxybutynin in 1988. Elderly patients or individuals with primary severe hyperhidrosis where surgery isn't eligible for them are turning progressively to this treatment as an alternate or initial therapy [21].

A case reported in 1988 described a patient with hyperhidrosis who began taking oxybutynin for urinary urgency; his episodes of severe sweating vanished within a few hours [22]. In addition, four other cases have been documented. From here, a hypothesis was generated about oxybutynin's promising benefits on patients with hyperhidrosis [23–25].

To our knowledge, no studies have made a meta-analysis on the use of oxybutynin in the treatment of hyperhidrosis, and due to the side effects of other mentioned treatments being significant, oxybutynin represents a possible alternative. So, this study aims to investigate patient satisfaction and the effectiveness of oxybutynin in treating hyperhidrosis.

## Methods

All the PRISMA statement guidelines were followed while reporting this systematic review and meta-analysis [26].

All steps have been done according to the Cochrane Handbook of Systematic Reviews and Meta-analysis of Interventions [27]. All steps of this study were prespecified, and the protocol was registered on PROSPERO (CRD42022342667).

### Eligibility criteria

Included studies in our review fit our inclusion criteria as the following:

**Population:** studies with patients who have hyperhidrosis or exercise-induced hyperhidrosis (physiologic sweating response to exercise).

**Intervention:** studies whose experimental group takes oral oxybutynin.

**Comparator:** studies where the control group received a placebo.

**Outcome:** studies that reported the transepidermal water loss, Hyperhidrosis disease severity scale, quality of life, dry mouth, and CNS adverse effects.

**Study design:** randomized studies that compare the oxybutynin group versus a placebo group.

We excluded studies reported as abstract only, review articles, observational studies, letters to the editor, comments, case reports, and studies that were not published in the English language.

### Information sources and search strategy

We performed a comprehensive search of three electronic databases (PubMed, Scopus, Web of Science) from inception until 2 June 2022 using MeSH terms. Further, the references of the included studies were manually searched for any potentially eligible studies. The search strategy and results for each database are reported in (supplementary material 1).

### Selection process

We removed duplicates by Endnote (Clarivate Analytics, PA, USA), and other records were screened independently by three authors in two steps: (1) title and abstract screening to determine the relevance to this meta-analysis, (2) full-text screening for the final eligibility to meta-analysis.

### Data collection process and data items

Data were collected independently by four review authors and extracted into a uniform data extraction Excel sheet. The extracted data included (1) Characteristics of the included studies, (2) Characteristics of the population of included studies, (3) Risk of bias domains, and (4) Outcome measures. Any disagreement between the review authors was resolved by consensus or consultation.

### Assessing the risk of bias in the individual studies

We used the Cochrane assessment tool 2 (ROB2) for randomized controlled trials. The risk of bias assessment included the following domains: bias arising from the randomization process, bias due to deviations from intended interventions, bias due to missing outcome data, bias in the measurement of the outcome, bias in the selection of the reported result, and other biases. The authors' judgments are categorized as "low risk," "high risk," or "some concerns" of bias [28].

### Synthesis methods

The risk ratio was calculated for categorical variables such as adverse effects to estimate the effect size and compare oxybutynin and placebo groups.

For continuous variables such as transepidermal water loss, the mean difference was calculated to estimate the effect size to assess the difference in outcome measures between oxybutynin and placebo groups.

### Choice of the meta-analysis model

We calculated the pooled effect size for all outcomes according to the DerSimonian Liard meta-analysis model. This random effect model assumes the included studies represent a random sample from the population and assigns a slightly higher weight to small studies on the expenses of larger studies. We chose this model because, unlike the fixed-effects model, it accommodates a larger standard error in the pooled estimate, which makes it suitable in case of inconsistent or controversial estimates. Thus, the calculated effects in our meta-analysis are conservative estimates that take into consideration the possible inconsistencies.

### Assessment of heterogeneity

Heterogeneity among studies was assessed using the Chi-square test (Cochrane *Q* test) and the *I*-squared (*I*^2^) and *χ*^2^ tests. *χ*^2^ *p*-value of < 0.1 indicates significant heterogeneity. *I*-square values ≥ 50% indicate high heterogeneity.

### Reporting bias assessment

In the present study, we could not assess the existence of publication bias by Egger’s test for funnel plot asymmetry, as according to Egger and colleagues[29], publication bias assessment is unreliable for < 10 pooled studies.

### Sensitivity analysis

To test the robustness of the evidence, we conducted a certainty assessment through sensitivity analysis (also called leave-one-out meta-analysis). We ran sensitivity analysis in multiple scenarios for every outcome in the meta-analysis, excluding one study in each scenario to ensure the overall effect size was not dependent on any single study.

## Results

### Literature search results

**Fig. 1:**
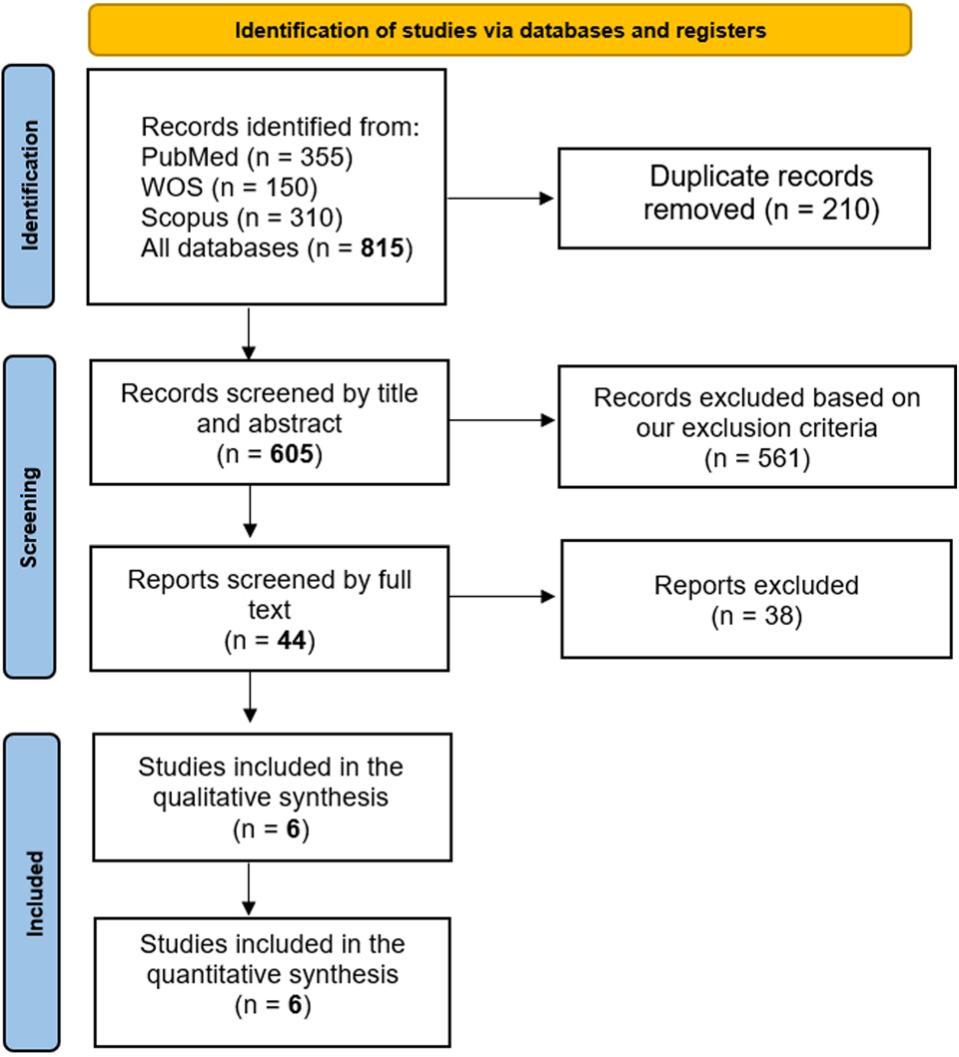
PRISMA flow diagram of studies’ screening and selection

Our literature search process retrieved 815 records. Following title and abstract screening, 44 articles were eligible for full-text screening. Of them, six studies were included in the meta-analysis. The references of the included studies were manually searched, and no further articles were included. The PRISMA flow diagram of the study selection process is shown in Fig. 1.

### Characteristics of the included studies

**Fig. 2:**
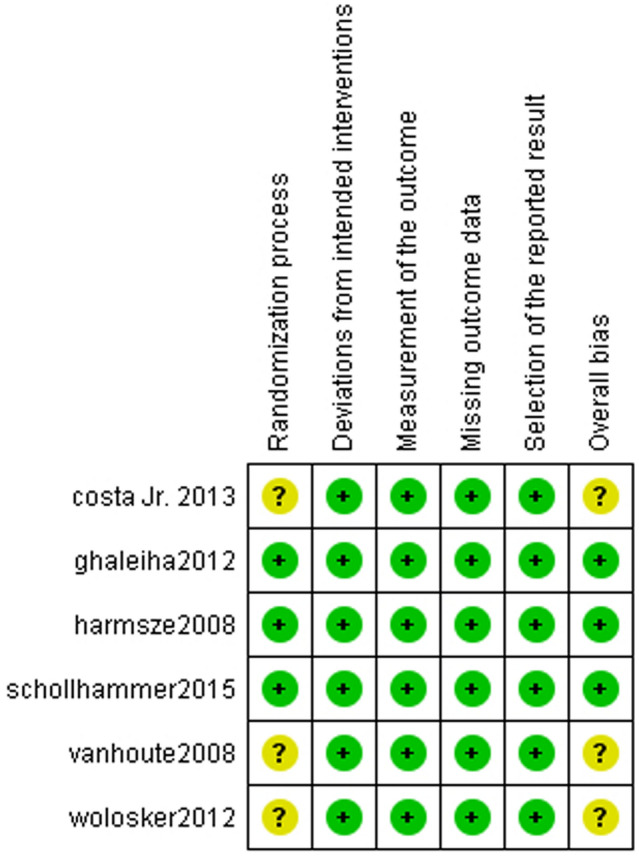
The risk of bias assessment

Six studies were included in the meta-analysis, with a total of 293 patients. In all studies, patients were assigned to receive either Oxybutynin or Placebo. A summary and baseline of the characteristics of the included studies are provided in Tables 1 and 2. Overall, the risk of bias in the included studies ranged from some concerns to low, according to the Cochrane risk of bias tool 2 (ROB2) (Fig. 2, and supplementary file sec “Methods”).

**Table 1:** Summary of the studies included in this systematic review and meta-analysis

Study ID	Title	Design	Country	Groups	Dose	No. of Participants	Population		Type of HH	Site of HH	Outcomes (Main results)
							Inclusion criteria	Exclusion criteria			
Ghaleiha [27]	Oxybutynin reduces sweating in depressed patients treated with sertraline: a double-blind, placebo-controlled, clinical study	RCT	Iran	Oxybutynin group	5 mg	66	Written informed consent from participants that were depressed and treated with 50 – 100 mg of sertraline for 14 days or more without any other disorders and physically healthy	Participants that Refused to provide their informed consent were diagnosed as substance abusers or with psychiatric disorders also who couldn’t tolerate the side effects of the placebo or the oxybutynin	Secondary (sweating related to sertraline use): 140(100%)	Whole body: 37 (60.7%), head and neck: 12(33.3%), axilla: 8(40%), palms: 2(33.3%)	Over time, oxybutynin decreased subjective sweating more than placebo
				Placebo group		74				Whole body: 24 (39.3%), head and nexk: 24(66.7%), axillia: 12(60%), palms: 4(66.7%)	
Schollhammer [28]	Oxybutynin as a treatment for generalized hyperhidrosis: a randomized, placebo-controlled trial	Prospective, randomized, placebo-controlled trial	France	Oxybutynin group	day 1 to 4: 2.5 mg, day 5 to 7: 5 mg, day 8 to the end of the six weeks: 7.5 mg	30	Written informed consent from participants that diagnosed as localized or generalized primary scoring 2 or above on the Disease Severity Scale (HDSS) with age equal to or above 18 years	Participants that Refused to provide their informed consent, aged below 18 years, the patient can’t be followed or in another trial, pregnant or breastfeeding women Any disease or therapies alternate oxybutynin activity	localized: 5 (17%), generalized: 25(83%)	Palmar: 22 (69%), planter: 22 (69%), axillary 24 (75%), fascial: 7 (22%), truncal 13 (41%)	Oxybutynin is superior to placebo in symptomatic relief as a treatment of localized and generalized hyperhidrosis. It also improves the quality of life with few adverse effects, such as dry mouth
				Placebo group		32			localized: 5 (17%), generalized: 25(83%)	Palmar: 14 (47%), planter: 17 (57%), axillary 21 (70%), fascial: 12 (40%), truncal 13 (43%)	
Vanhoute [29]	Effect of oxybutynin on exercise-induced sweating in healthy individuals	Two randomized, double-blind, placebo-controlled, cross-over studies	Netherland	Oxybutynin group	2.5 mg and 5 mg	8	healthy volunteers	Individuals below 18 and above 50 years, Any disease or medications alternate oxybutynin activity	exercise-induced sweating (normohidrotic individuals)	No HH	There is no significant difference between the two groups at all dosages of oxybutynin at the level of the forearm and the hand
				Placebo group							
Wolosker [2]	A randomized placebo-controlled trial of oxybutynin for the initial treatment of palmar and axillary hyperhidrosis	A prospective, randomized, controlled study	Brazil	Oxybutynin group	day 1 to 7: 2.5 mg once daily, day 8 to 21: 2.5 mg twice daily, day 22 to the end of 6 weeks: 5 mg twice daily	23	Patients with palmar or axillary hyperhidrosis intended to take other therapies to avoid oxybutynin side effects	Any disease or medications alternate oxybutynin activity	Focal: 45 (100%)	palmar: 11 (47.8%), axillary 12 (52.1%)	The improvement in quality of life (QOL) after treatment was significant much in the oxybutynin group. Oxybutynin usage is a good initial replacement therapy for hyperhidrosis
				Placebo group		22				palmar: 10 (45.4%), axillary 12 (45.5%)	
Harmsze [30]	Exercise-induced Sweating in Healthy Subjects as a Model to Predict a Drug's Sweat-reducing Properties in Hyperhidrosis: a Prospective, Placebo-controlled, Double-blind Study	Randomized, double-blind, placebo-controlled, cross-over study	Netherland	Oxybutynin group	5 mg (participants were given four tabs before an evaluation)	8	healthy volunteers	individuals with any diseases or therapies that affect oxybutynin activity	exercise-induced sweating (normohidrotic individuals)	No HH	There is no significant difference between the two groups at all exercise levels according to transepidermal water loss
				Placebo group							
Costa Jr [31]	Randomized trial—oxybutynin for treatment of persistent plantar hyperhidrosis in women after sympathectomy	RCT double-blind with a control arm	Brazil	Oxybutynin group	2.5 up to 10 mg	16	female suffered from plantar hyperhidrosis for more than six months and underwent G3 and G4 thoracic sympathectomy	Pregnant or breastfeeding women, females suffering from glaucoma, patients who used tricyclic drugs, obese patients and who used anticholinergic drugs	NR	planter: 32 (100%)	Oxybutynin is powerful and secure for women with chronic plantar hyperhidrosis who had undergone thoracic sympathectomy
				Placebo group		16

**Table 2:** Baseline characteristics of the included studies

Study ID	Groups	No. of participants	Age	Sex (Males)	BMI	Hyperhidrosis severity scale (HDSS)	Dermatology Life Quality Index (DLQI) score	
			Mean (SD)	N (%)	Mean (SD)		Mean	SD
Ghaleiha [27]	Oxybutynin group	66	37.12(11.47)	27(40.9)	25.7(4.61)	Grade 1: 0(0), grade 2: 27(40.9%), grade 3: 21(31.8%), grade 4: 18(27.3%)	NR	
	Placebo group	74	38.27(9.47)	27(36.4)	25.63(6.09)	Grade 1: 0(0), grade 2: 38(51.4%), grade 3: 24(32.4%), grade 4: 12(16.2%)		
Schollhammer [28]	Oxybutynin group	30	34.3(11.3)	11(37)	NR	Grade 2: 3(10%), grade 3: 17(57%), grade 4: 10(33%)	11.4	4.1
	Placebo group	30	36.4(12.3)	15(50)		Grade 2: 2(7%), grade 3: 18(60%), grade 4: 10(33%)	10.8	4.7
Vanhoute [29]	Oxybutynin group	8	28.5(7.94)	4(50)	NR	NR	NR	
	Placebo group							
Wolosker [2]	Oxybutynin group	23	28.4(9.4)	6(26.08)	NR	NR	Quality of life score: [84–100] (very poor): 15 (65.2%), [68–83] (poor): 8 (34.8%), [52–67] (good): 0 (0%), [36–51] (very good) 0 (0%), [20–35] (excellent) 0 (0%)	
	Placebo group	22	28(9)	6(27.2)			Quality of life score: [84–100] (very poor): 16 (72.7%), [68–83] (poor): 6 (27.3%), [52–67] (good): 0 (0%), [36–51] (very good) 0 (0%), [20–35] (excellent) 0 (0%)	
Harmsze [30]	Oxybutynin group	8	35.5(8.08)	NR	NR	NR	NR	
	Placebo group							
Costa Jr [31]	Oxybutynin group	16	28(6.4)	NR	22.4(2.1)	NR	NR	
	Placebo group	16	25.5(6.1)		21.3(1.3)

### Transepidermal water loss (TEWL)

#### TEWL for hand

**Fig. 3:**
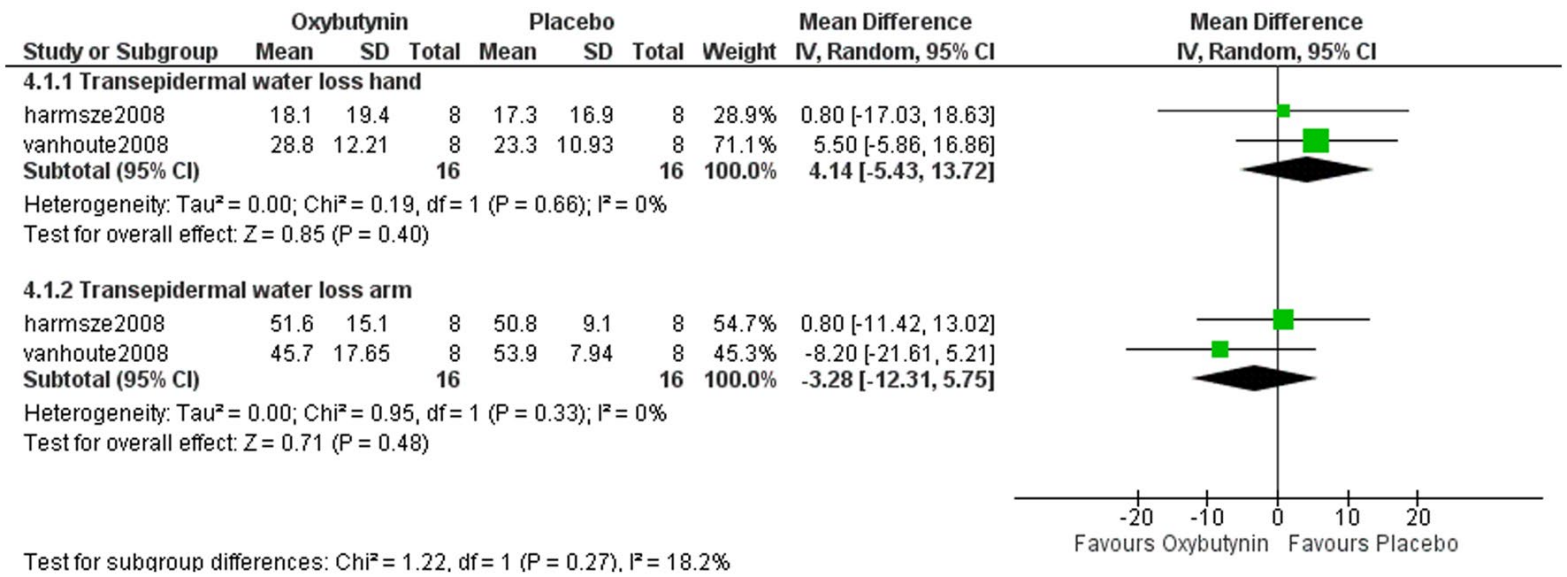
The comparison between oxybutynin and placebo according to transepidermal water loss

Two studies assessed TEWL for hand, involving 32 patients. The overall analysis showed non-statistically significant differences between oxybutynin and placebo according to TEWL for hand (MD = 4.14, 95% CI = [− 5.43 to 13.72], *P* = 0.4). Pooled studies were homogenous (*P* = 0.66, *I*^2^ = 0%) Fig. 3.

#### TEWL for arm

Two studies represented TEWL for arm, including 32 patients. The overall analysis showed non-statistically significant differences between oxybutynin and placebo according to TEWL for arm (MD = − 3.28, 95% CI = [− 12.31 to 5.75], *P* = 0.48). Pooled studies were homogenous (*P* = 0.33, *I*^2^ = 0%) Fig. 3.

#### Other sites

We found Costa et al. [30] representing this outcome at different sites in the body but unfortunately, we couldn’t pool the results in a meta-analysis. It measures TEWL on the back, right foot, right hand, and abdomen. Results showed that oxybutynin significantly reduces the TEWL. The mean (SD) change of the back was − 27.4 (64.8), 0.8 (33.75), of the abdomen, was − 23.2 (49.8), 2.8 (36,2), of the right hand, was − 33.1 (48.4), − 7.9 (54.5), of the right foot was − 52.7 (80.9), − 10.4 (74.5) in the oxybutynin group and in the placebo group respectively.

### Hyperhidrosis disease severity scale (HDSS) improvement

**Fig. 4:**
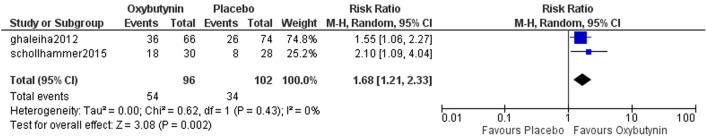
The comparison between oxybutynin and placebo according to Hyperhidrosis Disease Severity Scale (HDSS) improvement

Two studies reported HDSS improvement involving 198 participants. The overall RR showed a significant difference between the two groups favoring the oxybutynin group (RR = 1.68 95% CI [1.21, 2.33], *p* = 0.002). The pooled studies were homogenous (*p* = 0.43, *I*^2^ = 0%) Fig. 4.

### Quality of life (QoL)

Two studies reported on the effect of oxybutynin on QoL; however, because they used different methods, they could not be pooled in a meta-analysis. However, we observed that in Wolosker et al. [2] there were 8 (34.8%) patients in the oxybutynin group in comparison to 0 (0%) in the placebo group showed much better improvement, and 9 (39.1%) in contrast to 3 (13.6%) in the placebo group showed a little better improvement.

In Costa Jr et al., this outcome was measured by the QoL questionnaire for hyperhidrosis, in which the lower value, the better QoL. Before the intervention, it was 52.3 ± 11.5 ‘‘Good’’ and 47.8 ± 13.0 ‘‘Very Good’’ for oxybutynin and placebo groups, respectively. After the intervention, the oxybutynin group showed much improvement, 34.0 ± 9.5 ‘‘Excellent’’ than the placebo group, which was still in the same category, 46.5 ± 12.2 ‘‘Very Good’’.

### Dry mouth

**Fig. 5:**
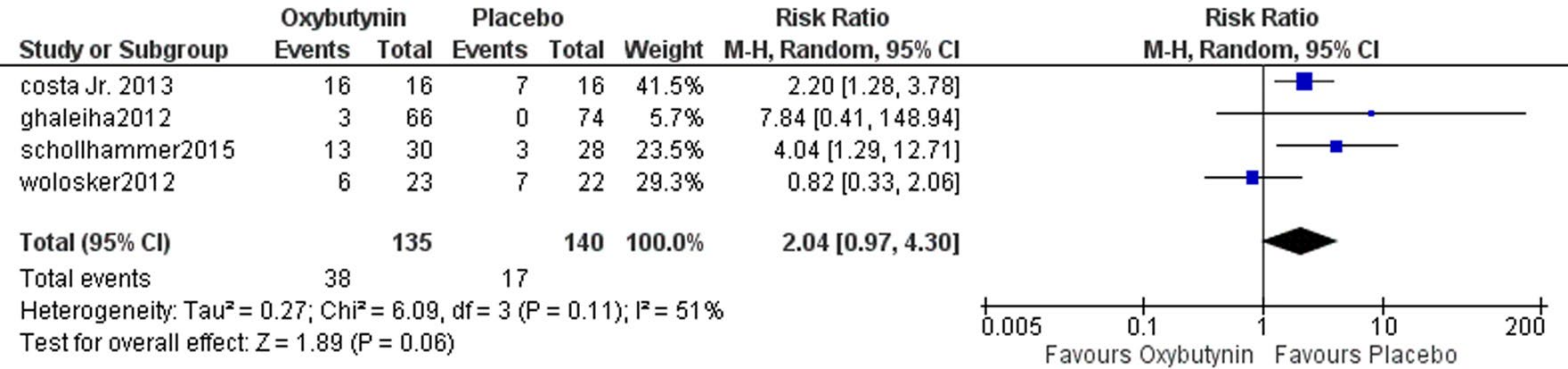
The comparison between oxybutynin and placebo according to dry mouth

Four studies assessed dry mouth involving 275 participants. The overall analysis showed non-statistically significant differences between oxybutynin and placebo according to dry mouth (RR = 2.04, 95% CI = [0.97 to 4.30], *P* = 0.06). The pooled studies were homogenous (*P* = 0.11, *I*^2^ = 51%) Fig. 5.

### ***Central nervous system (CNS) adverse effects (headache, asthenia, dizziness, flush, blurring vision, drowsiness, urinary difficulty)***

**Fig. 6:**
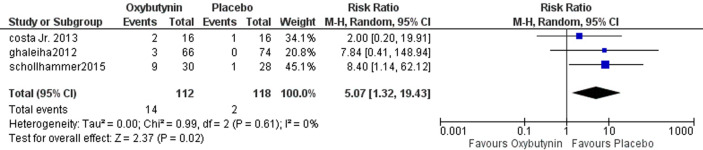
The comparison between oxybutynin and placebo according to CNS side effects

Three studies reported CNS adverse effects involving 230 patients. The overall analysis showed statistically significant differences between oxybutynin and placebo, favoring the placebo group according to CNS adverse effects (RR = 5.07, 95% CI = [1.32 to 19.43], *P* = 0.02). The pooled studies were homogenous (*P* = 0.61, *I*^2^ = 0%) Fig. 6.

## Discussion

### Summary of findings

As far as we know, no previous systematic reviews have been published to investigate the outcomes of oxybutynin treatment for patients with localized or generalized/ primary or secondary hyperhidrosis. The analysis includes four comparisons with 293 patients divided into the oxybutynin and the placebo groups. Our pooled analysis showed a significant difference between the oxybutynin and the placebo groups in HDSS improvement, favoring the oxybutynin group, and a clear difference in CNS adverse effects favoring the placebo group. The analysis showed no statistical difference between the two groups according to TEWL either on the hand or the arm and no apparent difference according to dry mouth.

We can’t analyze the quality of life as it was reported by two different methods in two studies. In both studies, oxybutynin showed improvement in the quality of life.

### Explanation of findings

Oxybutynin administration is associated with a low HDSS score, a decrease in the incidence of psychosocial functioning impairment, and an improvement in quality-of-life score. These effects contribute to the actual action of oxybutynin as it plays on the underlying mechanisms of HH. It contra verse acetylcholine receptors, especially muscarinic receptors M1, M2, and M3 [30]. By this antimuscarinic effect, it reduces sweating by limiting the role of acetylcholine on sweat glands. The non-statistical difference regarding TEWL does not necessarily deny the oxybutynin’s effectiveness, as the patients in the studies that measured the TEWL were healthy and did an exercise to induce hyperhidrosis.

A systematic review conducted by Cruddas et al. reported that anticholinergic therapy does not induce tachyphylaxis [31], which supports the effectiveness of oxybutynin in long-term usage [5]. It was reported that oxybutynin in the form of topical gel is the most commonly used anticholinergic as it provides a long duration of action extending to 84 h [32]. However, there is dreadful concern regarding oxybutynin since it is associated with CNS events that are favored by the ability to pass through the blood–brain barrier [13].

Adverse events from oral oxybutynin activity include dry eyes, mydriasis, headache, gastrointestinal symptoms, urinary retention, and orthostatic hypotension, but dry mouth is by far the most common side effect affecting 73.4%, 38.6%, and 68.8% of patients treated orally with oxybutynin [31]. However, our meta-analysis found no significant difference between the oxybutynin and placebo groups regarding the incidence of dry mouth. Despite the undesirable antimuscarinic effects of this medication, this drawback phenomenon can be prevented or even anticipated by using the stepwise up-titration approach that can achieve efficacy, tolerability, and therapeutic compliance, thus obtaining patient satisfaction [33]. If the patients can’t tolerate the side effects, many therapy modalities are available: oral, topical, and injectable drugs, tap water iontophoresis, or more invasive medical treatments (e.g., suction curettage, laser therapy, endoscopic transthoracic sympathectomy, and microwave thermolysis [13].

### Strength points

To date, this is the first meta-analysis to be published investigating the efficacy and safety of oxybutynin in patients with HH. Our research has several merits since it covers all the studies available in this vital area which gives our meta-analysis some strength. In addition, all included studies were randomized control trials that provide strong evidence.

### ***Limitations***

With the available data revised, we acknowledge that our meta-analysis has numerous limitations. For example, the number of eligible included studies in this meta-analysis is six, so we recommend more studies be conducted on the same vital topic. The difference in measuring each outcome makes it difficult to do the meta-analysis, and some studies were reported with us in systematic review only in some outcomes. No evaluation of patient satisfaction regards the drug's side effects and their impact on their quality of life. We can't make conclusions about the effectiveness of oxybutynin in patients with hyperhidrosis, as this study population was not exclusive to patients with hyperhidrosis and also included a healthy population.

### ***Recommendations***

We recommend further well-designed, high-quality, homogeneous RCTs with actually diseased patients with HH to investigate the efficacy and safety of oxybutynin. We recommend further evaluation of the impact of side effects of the drug on the patient's quality of life in future research, along with what should be done when the patients do not tolerate the drug. Also, future studies should compare the effect of the different treatment types.

Until the development of higher-quality evidence, we recommend the use of oxybutynin in patients with HH.

## Conclusion

Our study suggests that using oxybutynin is significant and needs to be highlighted for clinicians. However, more clinical trials are needed to grasp the optimum benefit.

## Supplementary Information

Below is the link to the electronic supplementary material.

Supplementary file1 (DOCX 23 KB)

## Data Availability

The datasets used and/or analyzed during the current study are available as MS Excel files (.xlsx) and RevMan file (.rm5) from the corresponding author upon reasonable request.

## Declarations

### Protocol registration

The protocol of this study was registered on PROSPERO (CRD 42022342667).
