# Supplementary material for: Safety and efficacy of oxybutynin in patients with hyperhidrosis: systematic review and meta-analysis of randomized controlled trials
Source: Arch Dermatol Res. 2023 Mar 4;315(8):2215–26. doi: 10.1007/s00403-023-02587-5 (PMC10462517; doi:10.1007/s00403-023-02587-5)
Supplement: Supplementary file 1 — Supplementary file1 (DOCX 23 KB) [file 403_2023_2587_MOESM1_ESM.docx]

## Search strategy for each database and its results:

**Pubmed:**

(Oxybutynin OR Ditropan OR Oxyb OR enamel OR Spasmex OR Tavor OR antispasmodics OR (Muscarinic Antagonists) OR anticholinergics) AND (Hyperhidrosis OR (excessive sweating))

Results: 355

**Web of science:**

(Oxybutynin OR Ditropan OR Oxyb OR enamel OR Spasmex OR Tavor OR antispasmodics OR “Muscarinic Antagonists” OR anticholinergics) AND (Hyperhidrosis OR "excessive sweating")

Results: 150

**Scopus**:

(Oxybutynin OR Ditropan OR Oxyb OR enamel OR Spasmex OR Tavor OR antispasmodics OR “Muscarinic Antagonists” OR anticholinergics) AND (Hyperhidrosis OR "excessive sweating")

Results: 310

## Risk of bias assessment of the included studies:

Table 1: Risk of bias according to the ROB 2 tool for the included studies

| Study ID | Randomization process | Deviations from intended interventions | Measurement of the outcome | Missing outcome data | Selection of the reported result | Overall bias |
| --- | --- | --- | --- | --- | --- | --- |
| Ghaleiha 2012 | Low risk | Low risk | Low risk | Low risk | Low risk | Low risk |
| Schollhammer 2015 | Low risk | Low risk | Low risk | Low risk | Low risk | Low risk |
| Van Houte 2008 | Some concerns | Low risk | Low risk | Low risk | Low risk | Some concerns |
| Wolosker 2012 | Some concerns | Low risk | Low risk | Low risk | Low risk | Some concerns |
| Harmsze 2008 | Low risk | Low risk | Low risk | Low risk | Low risk | Low risk |
| Costa Jr 2013 | Some concerns | Low risk | Low risk | Low risk | Low risk | Some concerns |


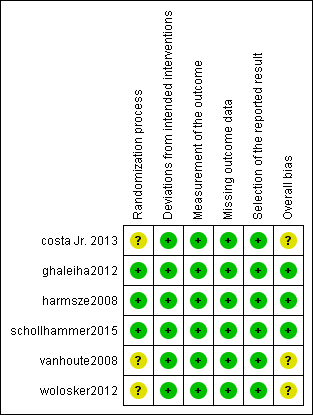


***Figure 1: show risk of bias summary***
